# A Polyamidoamine-Based Electrochemical Aptasensor for Sensitive Detection of Ochratoxin A

**DOI:** 10.3390/bios13110955

**Published:** 2023-10-26

**Authors:** Xiujin Chen, Dong Gao, Jiaqi Chen, Xueqing Wang, Chifang Peng, Hongli Gao, Yao Wang, Zhaozhou Li, Huawei Niu

**Affiliations:** 1 Hanan International Joint Laboratory of Food Green Processing and Quality Safety Control, National Demonstration Center for Experimental Food Processing and Safety Education, College of Food and Bioengineering, Henan University of Science and Technology, Luoyang 471000, China; 220320070680@stu.haust.edu.cn (D.G.); 210321090592@stu.haust.edu.cn (J.C.); 211416020101@stu.haust.edu.cn (X.W.); ghl3488579@haust.edu.cn (H.G.); wangyao@haust.edu.cn (Y.W.); lizhaozhou@haust.edu.cn (Z.L.); niuhuawei@haust.edu.cn (H.N.); 2 School of Food Science and Technology, Jiangnan University, Wuxi 214122, China

**Keywords:** Ochratoxin A, PAMAM, electrochemical aptasensor, graphene oxide, AuNPs

## Abstract

Sensitive detection of ochratoxin A (OTA) is significant and essential because OTA may pose risks to human and animal health. Here, we developed an electrochemical aptasensor for OTA analysis using polyamidoamine (PAMAM) dendrimers as a signal amplifier. As a carrier, PAMAM has numerous primary amino groups that can be coupled with thiolated complementary strand DNA (cDNA), allowing it to recognize aptamers bound to the surface of horseradish peroxidase (HRP)-modified gold nanoparticles (AuNPs), thereby improving the sensitivity of the aptasensor. When monitoring the positive samples, OTA was captured by the aptamer fixed on the HRP-conjugated AuNP surface by specific recognition, after which the formed OTA-aptamer conjugates were detached from the electrode surface, ultimately decreasing the electrochemical signal monitored by differential pulse voltammetry. The novel aptasensor achieved a broad linear detection range from 5 to 10^5^ ng L^−1^ with a low detection limit of 0.31 ng L^−1^. The proposed aptasensor was successfully applied for OTA analysis in red wine, with recovery rates ranging from 94.15 to 106%. Furthermore, the aptasensor also exhibited good specificity and storage stability. Therefore, the devised aptasensor represents a sensitive, practical and reliable tool for monitoring OTA in agricultural products, which can also be adapted to other mycotoxins.

## 1. Introduction

Ochratoxins are a group of common contaminants produced by molds from the genera *Aspergillus and Penicillium*. Among them, OTA has been identified as one of the most toxic and most widely distributed contaminates of agricultural products [1]. OTA is a stable molecule that remains intact under most processing conditions. Moreover, OTA poses many potential risks, including nephrotoxicity, hepatotoxicity, carcinogenicity, neurotoxicity, teratogenicity and immunotoxicity [2]. For these reasons, the European Union has established maximum permitted levels of OTA in dried fruits (10 μg kg^−1^), red wine (2 μg kg^−1^), beverages (2 μg kg^−1^) and processed cereals (3 μg kg^−1^) [3,4,5]. Thus, it is of great importance to develop a sensitive, accurate and facile approach for OTA detection. To date, a variety of analytical methods, including high-performance liquid chromatography with fluorescence detection (HPLC-FLD) [6] and liquid chromatography with mass spectrometry [7], have been reported for the detection of OTA in agricultural products, but these approaches require expensive equipment, trained personnel and complicated sample pretreatment, making them unsuitable for use in rural areas, where they are most needed. Subsequently, several rapid detection assays including gold-labeled immunochromatographic strips [8,9], magneto-gold nanohybrid-enhanced immunochromatographic strip [10] and enzyme-linked immunosorbent assay (ELISA) [11] have been developed, but all of these methods rely on the quality of the antibody used, whose production takes a long period of 6 months and requires tedious animal experiments. Additionally, the gold-labeled immunochromatographic strips have a low sensitivity due to the weak optical properties of gold nanoparticles [12]. Accordingly, it is crucial to establish a sensitive and feasible analytical approach for quantitative determination of OTA.

An aptamer is typically an RNA or single-stranded DNA composed of 20–80 nucleotides screened from a single-stranded nucleic acid library [13,14]. Compared with traditional antibodies, aptamers possess remarkable merits, such as easy chemical modification, high stability and a low cost. Currently, aptasensors are often integrated into electrochemical devices owing to their portability, rapidity and sensitivity [15]. Additionally, the performance of biosensors is not only related to the characteristics of the integrated aptamers but also the electrode-modifying nanomaterials. Accordingly, several nanomaterials are being utilized in the establishment of electrochemical aptasensors for the rapid determination of OTA in foodstuffs [16,17]. For example, Abnous’s group [18] designed an amperometric aptasensor for OTA analysis with carbon nanotubes as electrochemical signal amplifiers, which achieved a detection limit of 0.66 nM. Sun and colleagues [19] investigated a novel electrochemical aptasensor using aptamer–graphene oxide nanoprobes and DNase I, which achieved a detection limit of 52 pM. Lv’s group designed a switchable electrochemical hairpin–aptasensor for OTA analysis using the support interface combined with gold nanospheres (AuNS) and multiwalled carbon nanotubes (MWCNTs). This aptasensor achieved a detection limit of 1 ng L^−1^ [20]. Hou’s group established a sensitive electrochemical aptasensor with Nafion-stabilized functionalized multiwalled carbon nanotubes (f-MWCNTs) as signal enhancers. The limit of detection reached 1 ng L^−1^ for OTA [21]. However, the performance of these electrochemical sensors still cannot meet the detection requirements of OTA in food samples. To overcome this limitation, PAMAM dendrimers were introduced into electrochemical aptasensors for OTA analysis. PAMAM nanomaterials comprising branched chains of alkyl diamines and tertiary amines have distinct advantages, such as hydrophilicity, good biocompatibility, flexible structure, as well as high chemical and mechanical stability [22]. Due to the abundant amino groups on its periphery [23], the performance of electrochemical sensors can be enhanced by increasing the conjugation sites of recognition probes. The partially oxidized form of graphene, graphene oxide (GO), is dispersible in aqueous media, and oxygen-containing groups on its surface can be utilized to immobilize biomolecules via covalent bonds [24]. However, GO has a highly disrupted sp^2^ carbon lattice and functional groups, reducing its conductivity [25]. To overcome this, GO was converted into reduced graphene oxide (rGO) to improve its charge transfer ability [26,27,28].

In this work, an electrochemical aptasensor was developed to determinate OTA using PAMAM-GO nanocomposites as a signal amplifier. GO was converted into rGO via electrochemical reduction to improve the conductivity of the glassy carbon electrode (GCE). PAMAM dendrimers were used to enhance the electrochemical signal by binding more aptamer nanoprobes. The presence of OTA caused the release of aptamer-linked HRP-AuNPs from the electrode surface, thereby reducing the oxidation of hydroquinone to p-benzoquinone by H_2_O_2_ and ultimately generating a weak electrochemical signal. Under the optimal experimental conditions, the devised sensing platform exhibited a good detection performance and high recovery rates in red wines.

## 2. Materials and Methods

### 2.1. Reagents and Insturments

#### 2.1.1. Reagents

OTA, aflatoxin B1(AFB1), zearalenone (ZEN), fumonisin B1 (FB1), deoxynivalenol (DON), PAMAM dendrimers and HAuCl_4_·3H_2_O were obtained from Sigma-Aldrich (Shanghai, China). Tris (2-carboxyethyl) phosphine (TCEP), bovine serum albumin (BSA) and horseradish peroxidase (HRP) were obtained from Shanghai Aladdin Reagent Co., Ltd. (Shanghai, China). GO was purchased from Shanghai Yuanye Biotechnology Co., Ltd. (Shanghai, China). Hydroquinone and H_2_O_2_ were purchased from Shanghai Macklin Biochemical Co., Ltd. (Shanghai, China). The following were prepared by Sangon Biotech. Co., Ltd. (Shanghai, China):

- Aptamer: 5′-SH-GAT CGG GTG TGG GTG GCG TAA AGG GAG CAT CGG ACA-3′;
- cDNA: 5′-NH2-TGT CCG ATG CTC-3′.

#### 2.1.2. Instruments

All the electrochemical experiments were carried on a CHI 660E Electrochemical Workstation (CH Instruments, Shanghai, China). The three-electrode system was composed of a GCE as the working electrode, platinum wire as the auxiliary electrode and Ag/AgCl as the reference electrode. The UV–Vis absorption spectra were recorded with a UV-2600 spectrophotometer (Shimadzu, Kyoto, Japan). Transmission electron microscopy (TEM) images were obtained using an HT-7700 transmission electron microscopy (Hitachi, Tokyo, Japan). Zeta potential measurements were performed using a BeNano 180 Zeta Pro zeta potential analyzer (Bettersize, Dandong, China). The FT-IR spectra were obtained using a Nicolet 6700 spectroscopy instrument (Thermo Fisher Scientific, Waltham, MA, USA).

### 2.2. Preparation of the HRP-AuNPs-Aptamer Nanoprobe

AuNPs (10 ± 2 nm) were synthesized referring to a reported study [29]. Briefly, 1 mL of HAuCl_4_ (*W/V* = 1%) was diluted with 79 mL of ultrapure water in a flask and then heated at 60 °C for 30 min to form solutions A. Afterwards, 4 mL of sodium citrate solution (*W/V* = 1%), 0.1 mL of tannic acid (*W/V* = 1%), 0.1 mL of K_2_CO_3_ solution (2.5 × 10^−2^ mol L^−1^) and 15.8 mL ultrapure water were mixed in another flask and heated at 60 °C for 0.5 h to form solution B. Subsequently, solution A was mixed with solution B under high-speed stirring and heated at 60 °C for approximately 10 min until the color of the solution turned wine red. The solution was stored at 4 °C. The AuNP solution was analyzed using UV-vis spectroscopy, TEM and zeta potential.

Firstly, 2 mL of AuNP solution was centrifuged, and the supernatant was removed; then, the precipitate was resuspended with 1 mL of ultrapure water and mixed well. Next, K_2_CO_3_ solution (0.01 mol L^−1^) was utilized to adjust the pH of the AuNP solution to 9.0. Then, 50 μL of horseradish peroxidase (HRP, 1 mg mL^−1^) was dropped into AuNP solution and incubated for 3 h under stirring. Finally, the OTA aptamer activated by TCEP (1 mg mL^−1^) was reacted with the obtained HRP-AuNP solution at 4 °C for 24 h. The HRP-AuNP-aptamer nanoprobes were stored at 4 °C. The HRP-AuNPs and the HRP-AuNPs-aptamer nanoprobes characterized via UV-vis spectroscopy in the wavelength range of 400–800 nm and zeta potential.

### 2.3. Synthesis of GO-PAMAM

After mixing, 2 mL of PAMAM (50 μM) and 2 mL of GO solution (0.5 g L^−1^) containing 20 mM KOH reacted at 40 °C for 12 h under stirring. Next, 160 μL of H_2_SO_4_ solution (0.5 M) was added and reacted for another 30 min. Finally, the GO-PAMAM suspension was centrifuged, and the supernatant was discarded to remove free PAMAM and GO. After the precipitate was resuspended in ultrapure water, the resuspension solution was centrifuged, and the supernatant was removed. The obtained precipitate was stored at 4 °C. GO and GO-PAMAM nanocomposites were analyzed using TEM and EDS (energy-dispersive X-ray spectroscopy).

### 2.4. Fabrication of the Aptasensor

Before the measurements, the GCE was polished sequentially with Al_2_O_3_ powder of 0.3 μm and 0.05 μm particle size. Then, the electrode was rinsed via sonication using ultrapure water and ethanol (KQ3200DE ultrasonic cleaner, Kunshan Ultrasound Instrument Co., Ltd., Kunshan, China) and dried by airing.

Then, 10 μL of GO-PAMAM (1 g L^−1^) was dripped on the GCE and incubated for 12 h to obtain GO-PAMAM-GCE. Then, GO was reduced by cyclic voltammetry (CV) in the potential range of −1.7~0.1 V. The rGO-PAMAM-GCE was immersed in 100 μL of glutaraldehyde (*W/V* = 2.5%) for 60 min to activate the amino group on the electrode surface and then rinsed with PPBS (0.01 mol L^−1^ potassium–phosphate buffer solution pH 7.4). Subsequently, 10 μL of cDNA (10 μM) was added onto the modified electrode surface and reacted for 16 h to fix the cDNA onto the rGO-PAMAM-GCE via amino groups. After that the electrode surface was cleaned with PPBS to remove unreacted cDNA, and then 1% BSA was placed onto the surface of the electrode coated with rGO-PAMAM-cDNA for 1.5 h to block the remaining active sites. Next, 10 μL of HRP-AuNPs-aptamer conjugates were dripped onto the modified electrode surface and reacted for 1 h at 37 °C to allow hybridization between the aptamer with cDNA. Ultimately, the electrode surface was cleaned with PPBS to wash free HRP-AuNPs-aptamer conjugates.

The as-prepared HRP-AuNPs-aptamer-BSA-cDNA-rGO-PAMAM-GCE was incubated with a range of concentrations of OTA for 50 min. When the modified electrode was immersed in PPBS containing 3 mM hydroquinone and 1.5 mM H_2_O_2_, the electrochemical signal was measured via differential pulse voltammetry (DPV) in the potential range of −0.2 to 0.6 V.

### 2.5. Electrochemical Measurements

The electrochemical measurements of different modified electrodes were conducted via CV scanning from −0.2 to 0.6 V at a scan rate of 0.05 V.s^−1^ and electrochemical impedance spectroscopy (EIS) in the frequency range of 10^−1^ to 10^5^ Hz with an amplitude of 0.005 V in an electrolyte comprising 5 mM K_3_[Fe(CN)_6_]/K_4_[Fe(CN)_6_] (1:1) in 0.1 mol L^−1^ KCl and PPBS. The electrochemical signal was measured through DPV. The concentrations of OTA were related to the change in peak current (ΔI = I_0_ − Ip). The peak current measured in the blank solution was recorded as I_0_, and the peak current measured in the indicated concentration of OTA was recorded as Ip. All measurements were performed at ambient temperature. All experiments were always performed in triplicate.

### 2.6. Sample Pretreatment

To access the applicability of the prepared aptasensor, red wines purchased from a Dazhang supermarket were chosen to perform spiked experiments. The red wine samples were from the supermarket. Firstly, 20 mL of methanol was mixed with 2.5 mL of red wine in a 50 mL centrifuge tube to form solution A. Then, 0.5 g of CH_3_COONa and 2.0 g of anhydrous MgSO_4_ were added to solution A. After being shaken for 3 min and centrifuged at 8000 rpm for 10 min, the supernatant remained, and the lower layer was re-extracted again using the same procedure. The two supernatants were combined and evaporated to near dryness at 40 °C under vacuum. The remaining substance was added into 2 mL of methanol and then filtered using a microporous membrane of 0.22 μm. After pretreatment, the red wines were spiked with the indicated concentrations of OTA and measured using the aptasensors and HPLC-FLD.

## 3. Results and Discussions

### 3.1. Functional Principle of the Electrochemical Aptasensor

**Figure 1:**
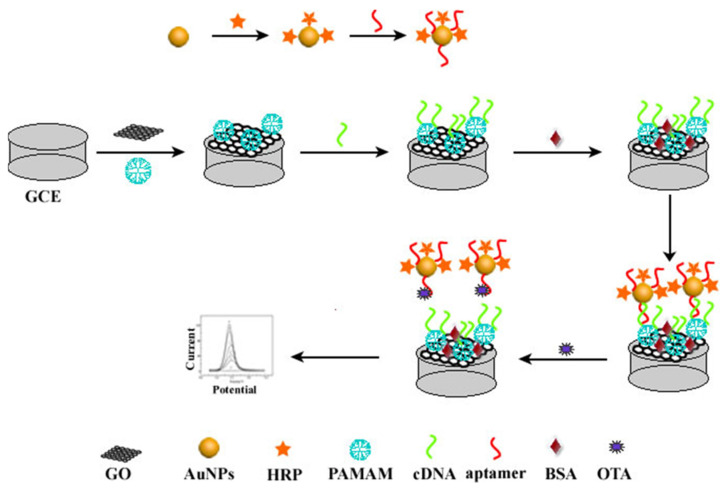
Schematic diagram of the electrochemical aptasensor.

The scheme of this proposed aptasensor based on HRP-AuNPs-aptamer nanoprobes and GO-PAMAM nanocomposites is shown in Figure 1. The GO-PAMAM nanocomposites were coated onto the surface of the electrode, which can not only enhance the surface electron transfer rate but also provide a great number of amino groups to capture cDNA molecules, thus enhancing the sensitivity of the electrochemical aptasensor. In the presence of OTA, aptamers will detach from the cDNA and capture OTA to form HRP-AuNPs-aptamer-OTA complexes, reducing the amount of fixed HRP-AuNPs-aptamer complex on the electrode surface. As a result, the electrochemical signal produced by HRP via H_2_O_2_ conversion decreased, and this change in electrochemical signal can be used for sensitive detection of OTA. At the same time, AuNPs in the HRP-AuNPs-aptamer nanoprobe can enhance the electrochemical signal by capturing greater numbers of aptamers and HRP molecules.

### 3.2. Characterization of the HRP-AuNPs-Aptamer Nanoprobe

**Figure 2:**
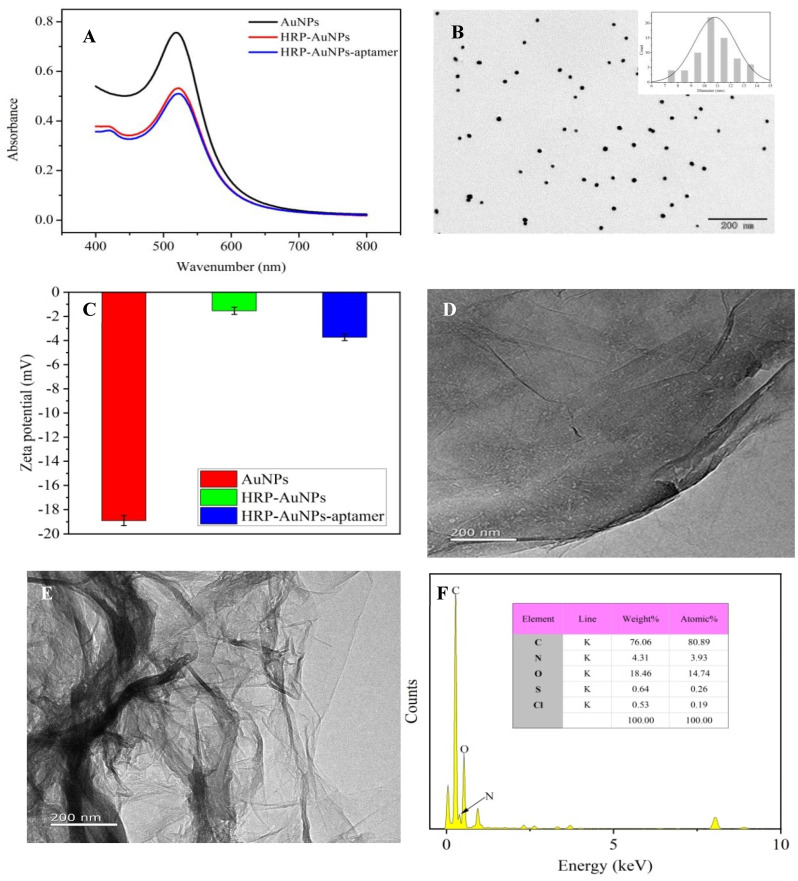
(**A**) UV−vis spectra of the AuNPs, HRP−AuNPs and HRP−AuNPs−aptamer; (**B**) TEM image of the AuNPs; (**C**) zeta potential of the AuNPs, HRP−AuNPs and HRP−AuNPs−aptamer (**D**) TEM image of the GO; (**E**) TEM image of the GO−PAMAM; (**F**) EDS analysis of GO−PAMAM.

The as-prepared AuNP solution was wine red. As illustrated in Figure 2A, the AuNP solution exhibited a strong absorption peak at 518 nm. When modified with HRP, the maximum absorption peak changed to 523 nm. When the aptamer was attached to HRP-AuNPs, the peak at 523 nm decreased, indicating that the coupling was successful (Figure 2A). This decrease was attributed to interparticle plasmon coupling. Under TEM observation (Figure 2B), the AuNPs were uniform and finely dispersed. The statistical analysis of the TEM data revealed that the average diameter of the prepared AuNPs was 10 nm. As revealed in Figure 2C, the surface of AuNP was modified with HRP via electrostatic interaction, which was demonstrated by the dramatic change in the zeta potential from −18.89 to −1.93 mV. After further reaction with the aptamer, the potential of the HRP-AuNP nanocomposite shifted to −4.09 mV, indicating the formation of the HRP-AuNPs-aptamer nanoprobe.

### 3.3. Characterization of GO-PAMAM

Amino groups in PAMAM reacted with carboxyl groups in GO to form GO-PAMAM conjugates. Firstly, the successful conjugation of GO-PAMAM was verified via TEM. As shown in Figure 2D, the surface of GO was smooth and corrugated, while that of GO-PAMAM (Figure 2E) was obviously wrinkled compared with that of GO alone. Energy-dispersive X-ray spectroscopy (EDS) of GO-PAMAM (Figure 2F) revealed the presence of 3.93% nitrogen atoms on its surface, which implied the successful synthesis of the GO-PAMAM nanocomposite.

**Figure 3:**
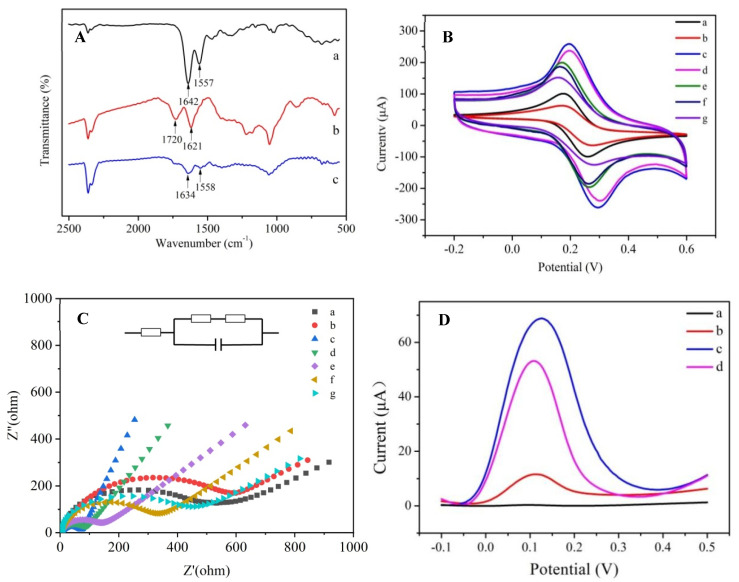
(**A**) FT−IR spectra of PAMAM (a), GO (b) and GO−PAMAM (c); (**B**) cyclic voltammograms of GCE modification, bare GCE (a), GO−PAMAM−GCE (b), rGO−PAMAM−GCE (c) cDNA−rGO−PAMAM−GCE (d), BSA−cDNA−rGO−PAMAM−GCE (e), HRP−AuNPs−aptamer−BSA−cDNA−rGO−PAMAM−GCE (f) and OTA−HRP−AuNPs−aptamer−BSA−cDNA−rGO−PAMAM−GCE (g); (**C**) electrochemical impedance spectra of GCE modification, bare GCE (a), GO−PAMAM−GCE (b), rGO−PAMAM−GCE (c), cDNA−rGO−PAMAM−GCE (d), BSA−cDNA−rGO−PAMAM−GCE (e), HRP−AuNPs−aptamer−BSA−cDNA−rGO−PAMAM−GCE (f) and OTA−HRP−AuNPs−aptamer−BSA−cDNA−rGO−PAMAM−GCE (g); (**D**) differential pulse voltammetry of the electrochemical aptasensor in PPBS (a) PPBS with 3 mM hydroquinone, (b) PPBS with 3 mM hydroquinone and 1.5 mM H_2_O_2_ (c) PPBS with 3 mM hydroquinone, 1.5 mM H_2_O_2_ and 100 ng/L OTA (d).

Additionally, Figure 3A shows the FT-IR spectra of PAMAM (a), GO (b) and GO-PAMAM (c). The FT-IR spectral peaks at 1557 and 1642 cm^−1^ correspond to the N-H bending vibration of secondary amide and C=O stretching vibration of primary amide in PAMAM, respectively (curve a). The FT-IR spectral peaks of GO at 1621 and 1720 cm^−1^ were assigned to the C=C stretching vibration of the aromatic ring and C=O stretching vibration of carboxyl, respectively (curve b). After PAMAM was coupled with GO, the FT-IR spectral peaks ascribed to the N-H bending vibration of the secondary amide and C=O stretching vibration of the primary amide could also be observed, but they shifted to 1558 and 1634 cm^−1^, respectively (curve c), which proves that the coupling of GO-PAMAM was successful [30]. Taken together, these results confirm that GO successfully reacted with PAMAM.

### 3.4. Characterization of the Modified Electrodes

The modification of the electrode was confirmed with cyclic voltammetry (CV). The results are illustrated in Figure 3B. Curve a exhibits two reversible redox peaks of the bare electrode. The peak current of the bare GCE became lower after modification with GO-PAMAM (curve b), which was attributed to the fact that PAMAM on the surface of GO reduces its conductivity. When GO was reduced, the peak current became stronger (curve c), as the active groups on the surface of GO were reduced, increasing its conductivity. After cDNA was attached to the surface of the GCE, the peak current became weaker owing to the poor conductivity of cDNA (curve d). After incubation with 1% BSA for 1 h, the peak current of the electrode further decreased (curve e). Although AuNPs have good conductivity, AuNPs loaded with HRP and aptamers could hinder the transfer of electrons on the electrode surface. After the electrode was incubated with the HRP-AuNPs-aptamer nanoprobe, the peak current of the GCE became lower (curve f). Ultimately, as OTA was incubated onto the surface of modified GCE, the aptamer and the cDNA can bind competitively to OTA. The electron transfer of Fe(CN)_6_^4−/3−^ ions on the GCE surface was blocked, and the peak current further decreased (curve g).

In addition, the modification of the electrode was also confirmed with electrochemical impedance spectroscopy (EIS). The EIS of the electrode is shown in Figure 3C. It can be seen from the figure that when the GO-PAMAM nanocomposite was dripped onto the electrode surface (635.5 Ω, curve b), the Rct increased compared with the bare electrode (378.4 Ω, curve a). This was caused by the poor conductivity of the GO-PAMAM nanocomposite, which decreased the electron transfer ability of the electrode surface. When the GO-PAMAM attached on the electrode surface was reduced to rGO-PAMAM, the Rct of the electrode surface was greatly reduced (74.64 Ω, curve c) owing to the good electron transfer ability of rGO. After the successive binding of cDNA (curve d) and BSA (curve e) onto the electrode surface, the Rct increased to 122.1 Ω and 183.6 Ω, respectively. This increase was attributed to the fact that electron transfer was hindered by cDNA and BSA. When the HRP-AuNPs-aptamer nanoprobe was attached to the electrode surface, the aptamer combined with cDNA to form a helical structure, which further hindered the electron transfer on the electrode surface, resulting in an increase in Rct (288.7 Ω, curve f). When the OTA sample was dripped onto the electrode surface, the HRP-AuNPs-aptamer nanoprobe was released, but a part of the OTA was still adsorbed onto the electrode surface, resulting in an increase in Rct (292.3 Ω, curve g). These results are consistent with those of the CV, confirming the successful surface modification of the electrode.

### 3.5. Electrochemical Properties of Modified Electrodes

The electrochemical performance of the aptasensor was studied using DPV. Figure 3D shows the DPV curves of the HRP-AuNPs-aptamer-BSA-cDNA-rGO-PAMAM-GCE electrode in the PPBS (0.01 mol L^−1^ potassium–phosphate buffer solution pH 7.4) (curve a), 0.01 mol L^−1^ PPBS containing 3.0 mM hydroquinone (curve b), PPBS containing 3.0 mM hydroquinone and 1.5 mM H_2_O_2_ (curve c) were recorded, respectively. The electrode did not produce an obvious reduction current peak in PPBS and PPBS containing 3.0 mM hydroquinone (curve b in Figure 3D), but the peak current increased dramatically when H_2_O_2_ was added (curve c), which was due to the oxidation of hydroquinone by HRP with H_2_O_2_ as co-substrate. When OTA was added, the DPV peak current decreased (curve d), indicating that OTA bound to the aptamer.

### 3.6. Optimization of the Assay Conditons for the Aptasensor

**Figure 4:**
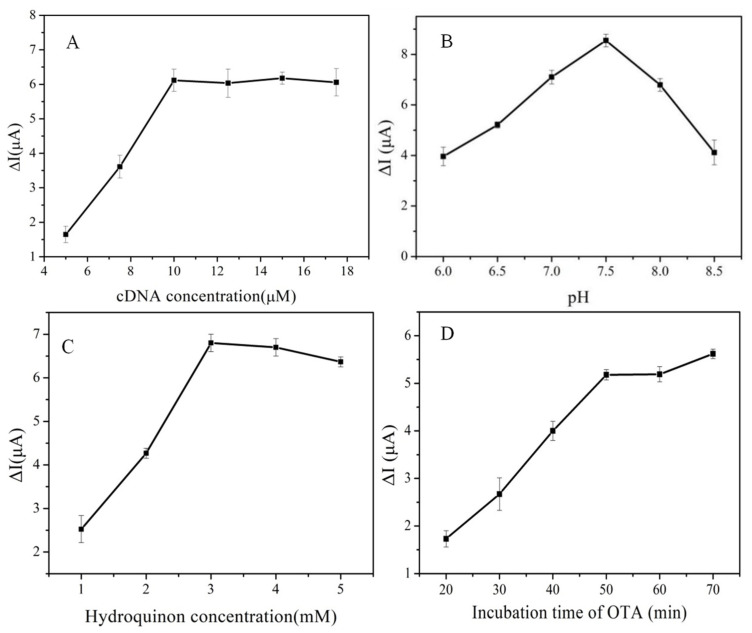
(**A**) The optimization of cDNA concentration; (**B**) pH; (**C**) hydroquinone concentration; (**D**) incubation time of OTA.

To optimize the performance of the designed aptasensor for OTA analysis, the cDNA concentration, pH, hydroquinone concentration and incubation time of OTA were optimized. Figure 4A shows the optimization results of cDNA concentration. The change in peak current went up with increasing cDNA concentration from 5 μmol L^−1^. The maximum change in peak current was achieved with a cDNA concentration of 10 μmol L^−1^. Later, the change in peak current tended to remain stable as cDNA concentrations were increased continuously. These results indicate the fixed amount of cDNA reached saturation when the concentration of cDNA was 10 μmol L^−1^. Hence, 10 μmol L^−1^ was chosen as the optimal concentration of cDNA. As seen from Figure 4B, when the pH of the assay solution was increased in the range of 6.0–8.5, the change in peak current increased first and then decreased, showing that the reaction system was too acidic or too alkaline, which is not conducive to the oxidation of hydroquinone. When the pH of the assay solution was 7.5, the change in peak current reached the maximum. Therefore, 7.5 was selected as the optimal pH. Figure 4C shows the influence of the hydroquinone concentration in the assay solution on the peak current change. The change in peak current rose first and then stabilized with the increase in hydroquinone concentration. This increase could be attributed to the fact that hydroquinone is the substrate of HRP, which exhibits Michaelis–Menten kinetics. When the hydroquinone concentration was 3 mmol L^−1^, the change in peak current reached the maximum value, followed by a plateau as the hydroquinone concentration exceeded 3 mmol L^−1^. Accordingly, the optimal concentration of hydroquinone was 3.0 mM. As the incubation time of OTA reached 50 min, the change in peak current remained almost unchanged (see Figure 4D), which indicated that the HRP-AuNPs-aptamer nanoprobe captured all of the available OTA. Hence, the optimal incubation time of OTA was determined to be 50 min.

### 3.7. Performance of the Proposed Aptasensor

**Figure 5:**
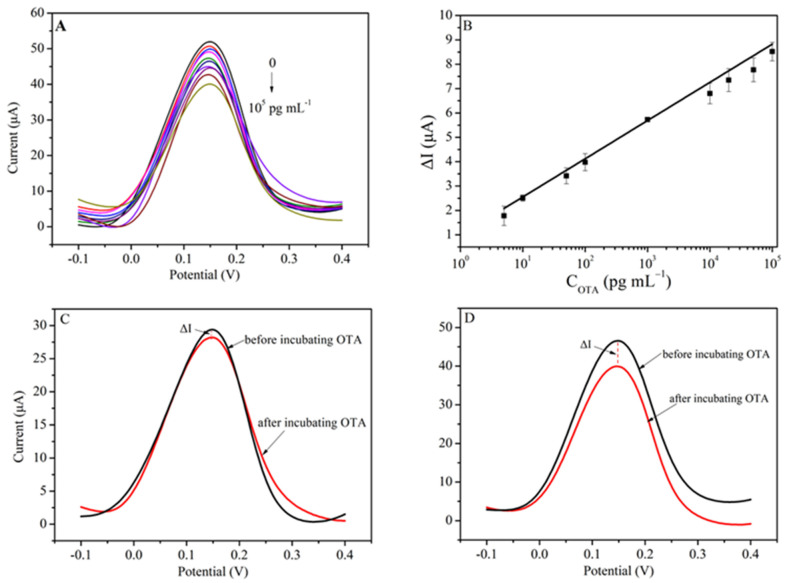
(**A**) Electrochemical signal response of the aptasensor to a series of concentrations of OTA; (**B**) the linear fit plot between ΔI and the logarithm of OTA concentrations; (**C**) DPV responses of the aptasensor without PAMAM modification; (**D**) DPV responses of the aptasensor with PAMAM modification.

Under the optimal conditions, the electrochemical aptasensor was applied for OTA detection at different concentrations (5, 10, 50, 10^2^, 10^3^, 10^4^, 2 × 10^4^, 5 × 10^4^, 10^5^ ng L^−1^) (seen in Figure 5A). The peak current change and the logarithm of OTA concentration range (5–10^5^ ng L^−1^) showed a good linear relationship (Figure 5B), and the linear equation was ΔI = 0.98387 + 1.56832 lg(C_OTA_) (r = 0.996). The grounds of the criterion (the detection limit (LOD) = 3δ/s), where δ was the standard deviation of the blank sample and the s was the slope of the calibration curve, the LOD was calculated as 0.31 ng L^−1^. In addition, PAMAM-GO conjugates were employed to improve the sensitivity of the electrochemical aptasensor. The reduction of GO on the electrode surface can amplify the electrical signal, and the use of PAMAM increases the available conjugation sites of the GCE, ultimately increasing the sensitivity of the biosensor. After incubation with 1.0 ng mL^−1^ OTA, the current changes of the aptasensor were tested using DPV and the ΔI increased from 1.78 to 5.63 μA, illustrating the signal amplification effect (Figure 5C,D). Compared with similar electrochemical aptasensors, the detection limit of the sensor developed in this study was lower (see Table 1).

### 3.8. Specificity, Stability and Reproducibility of the Aptasensor

**Figure 6:**
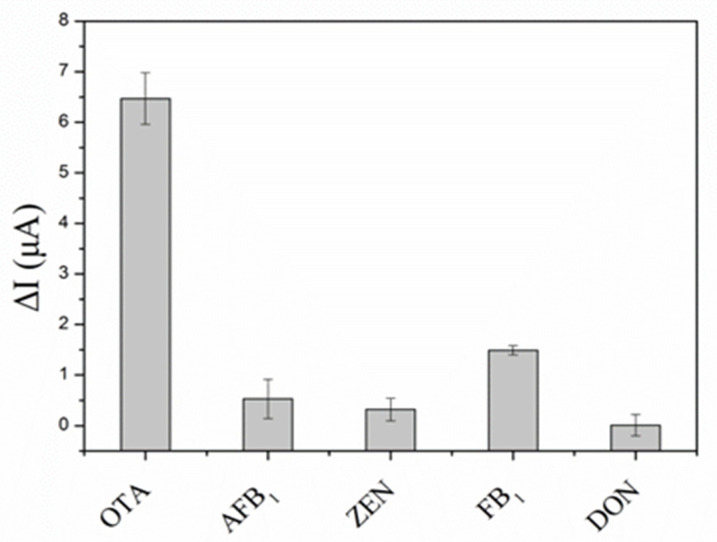
Specificity of the electrochemical aptasensor.

In order to evaluate the specificity of this method, four common mycotoxins, including aflatoxin B_1_ (AFB1), zearalenone (ZEN), fumonisin B1 (FB1) and deoxynivalenol (DON), were tested with the obtained aptasensor. The concentrations of OTA and the interfering mycotoxins were 1.0 and 10 µg L^−1^, respectively. As illustrated in Figure 6, when the biosensor was utilized to detect OTA, the peak current changed obviously. By contrast, although the concentrations of other mycotoxins were 10 times higher than the concentration of OTA, the current was practically unchanged. These results demonstrate that the aptasensor had good selectivity and specificity. To assess its stability, the prepared HRP-AuNPs-aptamer-cDNA-BSA-rGO-PAMAM-GCE was stored at 4 °C. After 21 days, the aptasensor retained 92.17% of the initial response current (Table S1), which proved that the aptasensor had high stability. In addition, the reproducibility of the assay was assessed in five parallel experiments with 1.0 µg L^−1^ OTA. These aptasensors exhibited highly similar current responses with a relative standard deviation (RSD) of 1.7% (Table S2), indicating that the aptasensor had good reproducibility.

### 3.9. Detection of OTA in Red Wine Samples

To assess the applicability of the aptasensor to real sample matrices, red wine samples were recovered with a series of OTA concentrations (5, 10, 50, 10^2^, 10^3^, 10^4^, 2 × 10^4^, 5 × 10^4^, 10^5^ ng L^−1^), pretreated and measured using the aptasensor under the optimized conditions. The prepared electrochemical aptasensor exhibited a good linear relationship with the OTA concentration from 5 to 10^5^ ng L^−1^. The linear equation was ΔI = 11.58483 + 3.01735 lg(C_OTA_) (r = 0.9980), with a detection limit of 0.47 ng L^−1^. The results obtained with the spiked samples are listed in Table 2. The recovery rates ranged from 94.15% to 106%, and the relative standard deviation (RSD) was 0.8–2.6%. Based on these results, the designed electrochemical aptasensor may be a competitive candidate for OTA determination in real samples with acceptable accuracy.

## 4. Conclusions

An electrochemical aptasensor was developed to detect OTA based on the dual amplification of PAMAM-GO nanocomposites and HRP-AuNPs-aptamer complexes as a nanoprobe. PAMAM was introduced to increase the number of binding sites for recognition probes while the reduced GO improved the conductivity of the modified GCE, realizing dual-signal amplification. Under the optimized assay conditions, the LOD of the proposed electrochemical aptasensor reached 0.31 ng L^−1^. Tests with spiked red wine samples showed recovery rates of 94.15–106%. Furthermore, the aptasensor also exhibited good specificity and stability. Compared with other similar electrochemical aptasensors, the established aptasensors exhibited an excellent electrochemical detection performance, which can be applied for highly sensitive detection of OTA in real food samples while also providing a promising platform for the determination of other small-molecule contaminants in food matrices.

## Figures and Tables

**Table 1:** Comparison of the proposed electrochemical aptasensor for the determination of OTA with previously reported sensors.

Nanomaterials	Linear Range (ng L^−1^)	LOD (ng L^−1^)	References
AuNPs	1–5 × 10^5^	1	[16]
Au nanopopcorns, Nafion-MWCNTs	1–10^4^	1	[17]
SWCNTs	80.6–1.81 × 10^4^	21	[18]
GO	0.01–5 × 10^4^	5.6	[19]
GNSs, MWCNTs	2–10^3^	1	[20]
f-MWCNTs	5–10^4^	1	[21]
PPy, PAMAM	2–6000	2	[22]
AuNPs	1–10^3^	0.5	[31]
AuNPs, MOF, MoS_2_	50–10^5^	10	[32]
AuNPs, GO	5–10^5^	0.31	This work

AuNPs: gold nanoparticles, Nafion-MWCNTs: Nafion-dispersed multiwalled carbon nanotubes, SWCNTs: single-walled carbon nanotubes, GO: graphene oxide, GNSs: gold nanospheres, MWCNTs: multiwalled carbon nanotubes, f-MWCNTs: functionalized multiwalled carbon nanotubes, PPy: polypyrrole, PAMAM: polyamidoamine, GO: graphene oxide, MOF: metal–organic framework.

**Table 2:** The detection of OTA in spiked red wine samples (*n* = 3).

Group	Added Level (μg L^−1^)	The Proposed Electrochemical Aptasensor			HPLC -FLD
		Found Level (μg L^−1^)	Recovery Rate (%)	RSD (%)	Found Level (μg L^−1^)
1	0.1	0.106	106	2.6	0.107
2	10	10.123	101.23	1.09	10.498
3	100	94.15	94.15	0.8	103.409

## Data Availability

The data presented are available on request from the corresponding author.

## Supplementary Materials

The following supporting information can be downloaded at: https://www.mdpi.com/article/10.3390/bios13110955/s1.

## References

[1] Nameghi M.A., Danesh N.M., Ramezani M., Hassani F.V., Abnous K., Taghdisi S.M. (2016). A fluorescent aptasensor based on a DNA pyramid nanostructure for ultrasensitive detection of ochratoxin A. Anal. Bioanal. Chem..

[2] Jiang C., Lan L., Yao Y., Zhao F., Ping J. (2018). Recent progress in application of nanomaterial-enabled biosensors for ochratoxin A detection. TrAC Trends Anal. Chem..

[3] Pagkali V., Petrou P.S., Salapatas A., Makarona E., Peters J., Haasnoot W., Jobst G., Economou A., Misiakos K., Raptis I. (2017). Detection of ochratoxin A in beer samples with a label-free monolithically integrated optoelectronic biosensor. J. Hazard. Mater..

[4] European Union (2006). Commission Regulation (EC) No 1881/2006 of 19 December 2006 setting maximum levels for certain contaminants in foodstuffs. Off. J. Eur. Union..

[5] Chen R., Sun Y., Huo B., Yuan S., Sun X., Zhang M., Yin N., Fan L., Yao W., Wang J. (2020). Highly sensitive detection of ochratoxin A based on bio-barcode immunoassay and catalytic hairpin assembly signal amplification. Talanta.

[6] Troestch J., Reyes S., Vega A. (2022). Determination of mycotoxin contamination levels in rice and dietary exposure assessment. J. Toxicol..

[7] Juan C., Mañes J., Juan-García A., Moltó J.C. (2022). Multimycotoxin analysis in oat, rice, almond and soy beverages by liquid chromatography-tandem mass spectrometry. Appl. Sci..

[8] Wang L., Ma W., Chen W., Liu L., Ma W., Zhu Y., Xu L., Kuang H., Xu C. (2011). An aptamer-based chromatographic strip assay for sensitive toxin semi-quantitative detection. Biosens. Bioelectron..

[9] Majdinasab M., Sheikh-Zeinoddin M., Soleimanian-Zad S., Li P., Zhang Q., Li X., Tang X. (2015). Ultrasensitive and quantitative gold nanoparticle-based immunochromatographic assay for detection of ochratoxin A in agro-products. J. Chromatogr. B Analyt. Technol. Biomed. Life Sci..

[10] Hao L., Chen J., Chen X., Ma T., Cai X., Duan H., Leng Y., Huang X., Xiong Y. (2021). A novel magneto-gold nanohybrid-enhanced lateral flow immunoassay for ultrasensitive and rapid detection of ochratoxin A in grape juice. Food Chem..

[11] Ren Y., Tian R., Wang T., Cao J., Li J., Deng A. (2023). An extremely highly sensitive ELISA in pg mL^−1^ level based on a newly produced monoclonal antibody for the detection of ochratoxin A in food samples. Molecules.

[12] Huang X., Aguilar Z.P., Xu H., Lai W., Xiong Y. (2016). Membrane-based lateral flow immunochromatographic strip with nanoparticles as reporters for detection: A review. Biosens. Bioelectron..

[13] Byun J. (2021). Recent progress and opportunities for nucleic acid aptamers. Life.

[14] Guan B., Zhang X. (2020). Aptamers as Versatile Ligands for Biomedical and Pharmaceutical Applications. Int. J. Nanomed..

[15] Chen X., Gao D., Sun F., Li Z., Wang Y., Qiu C., He K., Wang J. (2022). Nanomaterial-based aptamer biosensors for ochratoxin A detection: A review. Anal. Bioanal. Chem..

[16] Chen W., Yan C., Cheng L., Yao L., Xue F., Xu J. (2018). An ultrasensitive signal-on electrochemical aptasensor for ochratoxin A determination based on DNA controlled layer-by-layer assembly of dual gold nanoparticle conjugates. Biosens. Bioelectron..

[17] Hou Y., Long N., Xu Q., Li Y., Song P., Yang M., Wang J., Zhou L., Sheng P., Kong W. (2023). Development of a Nafion-MWCNTs and in-situ generated Au nanopopcorns dual-amplification electrochemical aptasensor for ultrasensitive detection of OTA. Food Chem..

[18] Abnous K., Danesh N.M., Alibolandi M., Ramezani M., Taghdisi S.M. (2017). Amperometric aptasensor for ochratoxin A based on the use of a gold electrode modified with aptamer, complementary DNA, SWCNTs and the redox marker Methylene Blue. Microchim. Acta.

[19] Sun A.L., Zhang Y.F., Sun G.P., Wang X.N., Tang D. (2017). Homogeneous electrochemical detection of ochratoxin A in foodstuff using aptamer-graphene oxide nanosheets and DNase I-based target recycling reaction. Biosens. Bioelectron..

[20] Lv L., Hu J., Chen Q., Xu M., Jing C., Wang X. (2022). A switchable electrochemical hairpin-aptasensor for ochratoxin A detection based on the double signal amplification effect of gold nanospheres. New J. Chem..

[21] Hou Y., Xu Q., Li Y., Long N., Li P., Wang J., Zhou L., Sheng P., Kong W. (2023). Ultrasensitive electrochemical aptasensor with Nafion-stabilized f-MWCNTs as signal enhancers for OTA detection. Bioelectrochemistry.

[22] Mejri-Omrani N., Miodek A., Zribi B., Marrakchi M., Hamdi M., Marty J.L., Korri-Youssoufi H. (2016). Direct detection of OTA by impedimetric aptasensor based on modified polypyrrole-dendrimers. Anal. Chim. Acta.

[23] Shen G., Xu H., Gurung A.S., Yang Y., Liu G. (2013). Lateral flow immunoassay with the signal enhanced by gold nanoparticle aggregates based on polyamidoamine dendrimer. Anal. Sci..

[24] Lan L., Yao Y., Ping J., Ying Y. (2017). Recent advances in nanomaterial-based biosensors for antibiotics detection. Biosens. Bioelectron..

[25] Malhotra B.D., Srivastava S., Ali M.A., Singh C. (2014). Nanomaterial-based biosensors for food toxin detection. Appl. Biochem. Biotechnol..

[26] Pei S., Cheng H.-M. (2012). The reduction of graphene oxide. Carbon.

[27] Pumera M., Ambrosi A., Bonanni A., Chng E.L.K., Poh H.L. (2010). Graphene for electrochemical sensing and biosensing. TrAC Trends Anal. Chem..

[28] Zhou M., Zhai Y., Dong S. (2009). Electrochemical sensing and biosensing platform based on chemically reduced graphene oxide. Anal. Chem..

[29] Khan A.K., Rashid R., Murtaza G., Zahra A. (2014). Gold Nanoparticles: Synthesis and Applications in Drug Delivery. Trop. J. Pharm. Res..

[30] Chen X., Wang Y., Zhang Y., Chen Z., Liu Y., Li Z., Li J. (2014). Sensitive electrochemical aptamer biosensor for dynamic cell surface N-glycan evaluation featuring multivalent recognition and signal amplification on a dendrimer-graphene electrode interface. Anal. Chem..

[31] Wang X., Shan Y., Gong M., Jin X., Jiang M., Xu J. (2019). A novel electrochemical sensor for ochratoxin A based on the hairpin aptamer and double report DNA via multiple signal amplification strategy. Sens. Actuators B Chem..

[32] Li D., Zhang X., Ma Y., Deng Y., Hu R., Yang Y. (2018). Preparation of an OTA aptasensor based on a metal–organic framework. Anal. Methods.

