# Supplementary material for: A Polyamidoamine-Based Electrochemical Aptasensor for Sensitive Detection of Ochratoxin A
Source: Biosensors (Basel). 2023 Oct 26;13(11):955. doi: 10.3390/bios13110955 (PMC10669513; doi:10.3390/bios13110955)
Supplement: Supplementary file 1 [file biosensors-13-00955-s001.zip › biosensors-2602685-supplementary.pdf]

Table S1 The retain rate of response current after 21 days

Group	Initial current(μA）	Final current (μA）	Retain rate	Average
1	57.82	53.35	92.27%	
2	55.23	50.78	91.94%	92.17%
3	54.23	50.06	92.31%	

Table S2 The assay was assessed in five parallel experiments with 1.0 ng mL^{-1} OTA

Group	OTA (ng mL^{-1})	Found level (ng mL^{-1})	Average	RSD
1		0.99		
2		0.98		
3	1.00	1.02	1.0	1.7%
4		1.10		
5		1.02		
